# Adult nutrition, but not inbreeding, affects male primary sexual traits in the leaf‐footed cactus bug *Narnia femorata* (Hemiptera: Coreidae)

**DOI:** 10.1002/ece3.2246

**Published:** 2016-06-16

**Authors:** Paul N. Joseph, Daniel A. Sasson, Pablo E. Allen, Ummat Somjee, Christine W. Miller

**Affiliations:** ^1^ Entomology and Nematology Department University of Florida 1881 Natural Area Drive Gainesville Florida 32608; ^2^ Whitney Laboratory for Marine Bioscience University of Florida 9505 Ocean Shore Blvd St. Augustine Florida 32080

**Keywords:** Adult nutrition, inbreeding, *Narnia femorata*, sperm concentration, sperm viability, testes

## Abstract

Adverse conditions may be the norm rather than the exception in natural populations. Many populations experience poor nutrition on a seasonal basis. Further, brief interludes of inbreeding can be common as population density fluctuates and because of habitat fragmentation. Here, we investigated the effects of poor nutrition and inbreeding on traits that can be very important to reproductive success and fitness in males: testes mass, sperm concentration, and sperm viability. Our study species was *Narnia femorata,* a species introduced to north‐central Florida in the 1950s. This species encounters regular, seasonal changes in diet that can have profound phenotypic effects on morphology and behavior. We generated inbred and outbred individuals through a single generation of full‐sibling mating or outcrossing, respectively. All juveniles were provided a natural, high‐quality diet of *Opuntia humifusa* cactus cladode with fruit until they reached adulthood. New adult males were put on a high‐ or low‐quality diet for at least 21 days before measurements were taken. As expected, the low‐quality diet led to significantly decreased testes mass in both inbred and outbred males, although there were surprisingly no detectable effects on sperm traits. We did not find evidence that inbreeding affected testes mass, sperm concentration, and sperm viability. Our results highlight the immediate and overwhelming effects of nutrition on testes mass, while suggesting that a single generation of inbreeding might not be detrimental for primary sexual traits in this particular population.

## Introduction

Male postcopulatory sexually selected traits, including testis size (*reviewed in* Parker 2016) and sperm traits (*reviewed in* Snook 2005), can be critical for sperm competition and reproductive success. However, environmental stressors may have detrimental effects on testes size and sperm quality (Gage and Cook 1994; Stockley and Seal 2001; Hellriegel and Blanckenhorn 2002; Engqvist 2008). Nutrition is one of the most important environmental factors influencing reproduction (*reviewed in* Awmack and Leather 2002; Katsuki et al. 2012). Individuals often experience environmental heterogeneity or seasonal fluctuations in their natural diet that lead to fluctuations in the size of testes and changes in sperm traits (Engqvist 2008; Lewis et al. 2012). While testis development (*reviewed in* Martin et al. 2012) and sperm traits (Rahman et al. 2014) are especially influenced by juvenile diet (Gage and Cook 1994; McGraw et al. 2007), the effects of adult diet on these traits remain unresolved (Hellriegel and Blanckenhorn 2002; Amitin and Pitnick 2007; Fricke et al. 2008; Bunning et al. 2015). For hemimetabolous insects specifically, adult diet is predicted to have a significant effect on sperm traits and testis growth as spermatogenesis continues through adulthood (Economopoulos and Gordon 1971; Dumser and Davey 1974).

Natural changes in nutritional quality likely do not occur in isolation, but are instead occurring against a backdrop of other stressors. Inbreeding is common in the wild (Armbruster et al. 2000) and can have detrimental effects on sperm quantity (Margulis and Walsh 2002; Weeks et al. 2009) and quality (Roldan et al. 1998; van Eldik et al. 2006; Gage et al. 2006; Asa et al. 2007; Fitzpatrick and Evans 2009).

Here, we investigate the effects of poor adult nutrition and inbreeding on testes mass, sperm concentration, and sperm viability in the leaf‐footed cactus bug *Narnia femorata*. Previous work has found *Narnia femorata* to be an excellent model to investigate the effects of natural diet variation on sexually selected and reproductive traits (Addesso et al. 2014; Gillespie et al. 2014). While previous work on this species revealed *juvenile* dietary effects on testes mass (Sasson et al. 2016), it is currently unknown whether poor *adult* diet combined with realistic levels of natural inbreeding also affect testis growth and whether they affect sperm traits. Based on work in other taxa and with this species, we predicted that outbred males with good adult nutrition would grow the largest testes and generate the most and highest viable sperm of all the treatments as they were free of these two stressors. We predicted that inbred males restricted to a poor adult diet would develop the smallest testes and would generate the fewest and least viable sperm. Our aim was to study the effects of poor nutrition and inbreeding using experimental manipulations that are rooted in the ecology of our species and likely to be relevant to natural situations. Thus, we use natural, seasonal differences in their cactus diet and a low level of inbreeding that may be routine in nature.

## Materials and Methods

### Study organism and host plant interactions

*Narnia femorata* is a phytophagous insect whose trait expression is influenced by seasonal variation in its host plant, the prickly pear cactus (*Opuntia* sp.). Ripe fruit availability varies seasonally and spatially due to prickly pear cactus phenology and due to interspecific competition for fruit (González‐Espinosa and Quintana‐Ascencio 1986; Hellgren 1994; Gillespie et al. 2014; L.A. Cirino et al. *in prep*.). In north‐central Florida, insects raised with ripe *Opuntia humifusa* fruit and cladodes develop more quickly (Nageon de Lestang and Miller 2009) and mature as larger, more attractive adults than conspecifics reared only on *O. humifusa* cladodes (Gillespie et al. 2014). Thus, cactus phenology serves as an ecologically relevant nutritional factor that is easily manipulated in laboratory settings to determine the effects of poor (cactus cladodes without fruit) and good (cactus cladodes with ripe fruit) diets. Males reared with ripe fruit have larger testes than males reared in the absence of fruit (Sasson et al. 2016). Variation in testis size, as seen in other taxa, may pose limitations to spermatogenesis (Møller 1988, 1989; Stockley et al. 1997). Thus, it is becoming well established that natural shifts in *juvenile* diet have measurable consequences for adult phenotypes in *N. femorata*. Here, we examine effects of early *adult* nutrition on primary sexual traits.

### Laboratory rearing

All the inbred and outbred individuals generated through this experiment were derived from 64 wild *N. femorata* individuals (the P generation) collected from the University of Florida's Ordway‐Swisher Biological Station in September 2014. Each individual was arbitrarily paired with another individual of the opposite sex. The pairs were housed in their own containers, consisting of soil substrate, a single *O. humifusa* cladode, and ripe fruit. Each pair was allowed to mate and oviposit freely. Hatched juveniles (the F1 generation) were subsequently moved to new cups, separate from their parents. These juveniles eventually served as the parents for our focal individuals.

### Experimental design

**Figure 1:**
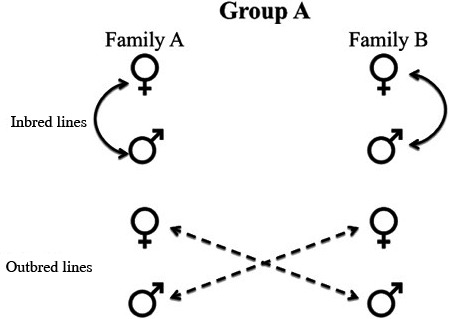
Illustration of the experimental design used. This design was created by Roff (1998) for testing phenotypic effects of inbreeding. Each group was composed of two families. Males and females from each family were crossed to establish outbred lines (dashed) while males and females from the same family were crossed to establish inbred lines (solid).

To test the effects of inbreeding and adult nutrition (i.e., access to good or poor nutrition) on male reproductive traits, we incorporated elements of the experimental design used by Roff (1998) to created inbred and outbred *N. femorata* (Fig. 1). We created 16 groups, each consisting of two P generation mating pairs (hereafter referred to as families). We mated the F1 offspring of one family either with siblings, creating inbred lines, or with the offspring from the corresponding family, creating outbred lines, for each group. As one family was crossed with only one other family, we controlled for the alleles within a given group (Roff 1998).

The F2 progenies generated through inbreeding and outcrossing were reared under similar laboratory conditions as the F1 parents. Once the inbred and outbred F2 individuals reached adulthood, each male was separated. Males were randomly assigned to one of two environmental treatments: good nutrition (cactus cladode with ripe fruit) or poor nutrition (cactus cladode without fruit). Individually housed adult males were subjected to the nutritional treatments for at least 21 days after adult eclosion, which was approximately 7 days after males reached reproductive maturity. Adult males were kept in their nutritional treatment until they were sacrificed. To reflect age differences in a natural population, we sacrificed males across a range of ages, between 21 and 73 days posteclosion. For this reason, we included adult male age as a covariate in some analyses. We obtained 50–51 males for each environmental/genetic treatment combination (representing 30 inbred lines and 22 outbred lines). Five groups did not produce outbred males. As one group only produced a single male, we excluded it from all analyses.

### Sperm analyses

We extracted fully mature sperm stored in both seminal vesicles of each male. These organs were suspended and ground in 200 *μ*L of HEPES‐buffered saline solution with 10% bovine serum albumin. We injected 10 *μ*L of the sperm–saline mixture into two chambers of a Hausser phase contrast hemocytometer. We counted the number of spermatozoa in a given area (1 mm^2^). As the depth was 0.1 mm, we multiplied this value by ten to calculate the number of spermatozoa/*μ*L. We did this twice per sample and then averaged the two concentrations.

Immediately following sperm concentration analysis, we used the remaining sperm from each male to calculate sperm viability. Following the guidelines provided by the live/dead sperm viability kit (Invitrogen, Molecular Probes), we combined 100 *μ*L of the remaining sperm–saline mixture with 0.5 *μ*L of 50‐fold diluted SYBR‐14 dye in a new microcentrifuge tube. After ten minutes of incubation at room temperature, we added 0.5 *μ*L of propidium iodide and incubated the mixture for an additional ten minutes. We then pipetted 10 *μ*L of the solution onto a slide with a cover slip and visualized the sperm using fluorescent microscopy. We counted between 100 and 250 total sperm cells per individual, depending on whether the sample was moderately or highly concentrated, and calculated the percentage of viable sperm. All sperm analyses were performed blind to experimental treatment.

### Testis mass and body size

We measured testis mass, as it is often correlated with sperm production and is often used as a measurement of sperm investment across species (Gage 1994, 1995; Parker and Ball 2005). Pairs of testes were dried at 40°C for 24 h in preweighed aluminum foil boats. We recorded the masses of testis pairs to the nearest microgram using a Sartorius Cubis Balance: Goettingen, Germany. We measured pronotum width to the nearest micrometer as an estimate of overall body size by first taking standardized photographs, using a Leica M165C stereomicroscope with an attached camera, and then using ImageJ software v. 1.46r (Abramoff et al. 2004). Previous work in this species has shown that this simple measurement provides an excellent estimate of overall size (Gillespie et al. 2014). Testis and body measurements were measured blind with respect to the experimental treatment.

### Group‐level statistical analyses

We first conducted statistical analyses at the group level; the group became the lowest level of independence for this set of analyses (Roff 1998). Five groups failed to generate outbred individuals; however, they were still included in the analyses comparing inbred males with different diets. We averaged the values of individuals according to treatment within each group. This resulted in 2–4 average values per group, with 15 groups.

We tested for significant differences in sperm concentration, sperm viability, and testes mass between the four treatments using generalized linear models (GLMs) with inbreeding status and adult diet each as an independent variable.

### Individual‐level statistical analyses

Next, we performed analyses that treated the individual as the lowest level of independence, disregarding group. Our goal was to begin to explore effects of inbreeding and nutrition while controlling for body size and the age of individuals. Individuals were not truly independent in this study, and some families had greater representation than others (likely pseudoreplication). The purpose was to provide the raw material for hypothesis generation, not to generate immediate conclusions (*see statistical methods in* Roff 1998). We used GLMs including pronotum width and the number of days as adult as covariates. Our response variables were the same as above: sperm concentration, sperm viability, and testes mass. The sperm concentration, testes mass, and pronotum width of individual males were not normally distributed when the individual was the lowest level of independence. Therefore, prior to analysis, we square‐root‐transformed sperm concentration and log‐transformed testes mass and pronotum width.

We constructed our individual‐level models using a backward elimination process (Hutcheson and Sofroniou 1999), where nonsignificant interactions were sequentially removed when *P *>* *0.10. No main effects were removed. We did not remove the inbreeding‐by‐nutrition interaction (although *P *>* *0.10, see Results) because testing this interaction was a motivation for the experiment. The models were finalized once interactions had *P ≤ *0.10. Therefore, there were interactions included in some models that were removed from others.

All group‐ and individual‐level analyses were performed using SPSS 21: Armonk, NY.

## Results

### Group‐level analyses

**Figure 2:**
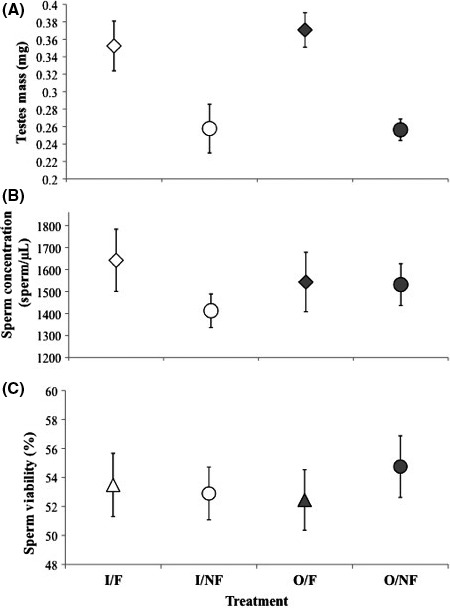
Mean and ± SE A) testes mass (mg), B) sperm concentration (sperm/*μ*L), and C) sperm viability (%) of inbred or outbred males with access good nutrition (fruit) and who were restricted to poor nutrition (no fruit). Males were either inbred and had access to fruit (I/F), inbred and had no access to fruit (I/NF), outbred and had access to fruit (O/F), or outbred and had no access to fruit (O/NF). Analyses were performed between the four treatments using the average values from 15 groups.

We found that poor nutrition had a large and negative effect on testes mass (Fig. 2A; Table 1), but inbreeding did not have a detectable effect. We did not find evidence that either inbreeding or poor nutrition affected sperm concentration (Fig. 2B; Table 1) or sperm viability (Fig. 2C; Table 1). We did not find an interaction between inbreeding and poor nutrition on any primary sexual trait (Table 1).

**Table 1:** Group‐ and individual‐level wald chi‐square values

	Testes mass		Sperm concentration		Sperm viability	
	Wald chi‐square	*P*	Wald chi‐square	*P*	Wald chi‐square	*P*
Group‐level analyses						
Nutrition	**18.443**	**<0.001**	1.229	0.268	0.182	0.670
Inbreeding	0.121	0.727	0.021	0.884	0.042	0.838
Nutrition*Inbreeding	0.165	0.684	0.792	0.373	0.516	0.472
Individual‐level analyses						
Nutrition	**15.426**	**<0.001**	0.224	0.636	0.820	0.365
Inbreeding	0.033	0.542	0.029	0.864	3.278	0.070
Nutrition*Inbreeding	0.116	0.734	0.767	0.381	0.003	0.953
Adult Age	**4.395**	**0.036**	**30.691**	**<0.001**	**16.466**	**<0.001**
Log Pronotum Width (PW)	2.446	0.118	**7.681**	**0.006**	0.001	0.971
Inbreeding*PW	–	–	–	–	3.218	0.073
Adult Age*PW	**5.246**	**0.022**	–	–	–	−

Bold values signify *P* < 0.05. Error degrees of freedom (df) for group‐level analyses = 48 (testes mass, sperm concentration, sperm viability).

Error degrees of freedom (df) for individual‐level analyses = 185 (testes mass), 189 (sperm concentration), 188 (sperm viability).

– = effect removed because *P *>* *0.10.

### Individual‐level analyses

These exploratory analyses are intended for hypothesis generation and should be interpreted with caution. Based on our GLM using backward elimination, we found that poor nutrition appears to have a significant negative effect on testes mass; however, poor nutrition did not have any detectable effects on sperm concentration or sperm viability (Table 1). Inbreeding did not have any detectable effects on testes mass, sperm concentration, or sperm viability (Table 1).

**Figure 3:**
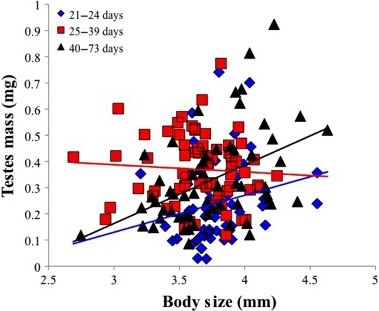
Interaction between body size (mm) and adult age (days) on testes mass (mg). Individuals were divided into three groups based on the number of days since adult eclosion: young (ages 21–24 days; *N* = 68), middle‐aged (ages 25–39 days; *N* = 68), and old (ages 40–73 days; *N* = 64). For the purpose of clarity in this figure, the data are pooled irrespective to diet and inbreeding status. Trend lines for each group (blue = 21–24 days, red = 25–39 days, black = 40–73) were estimated from the raw data.

Older adults may have greater testes mass and sperm concentration, but less viable sperm (Table 1). Larger males appear to generate higher sperm concentrations (Table 1). Finally, testes mass seems to increase with increasing body size, but only for males on the lower and upper ends of the age spectrum (Fig. 3).

## Discussion

We found that *Narnia femorata* provided with inferior nutrition as adults had smaller testes, a result that echoes findings on nutrition and testes development across taxa (*reviewed in* Martin et al. 2012; *also see* Genc and Nation 2004; Butler et al. 2013). To our knowledge, this is the first study to show natural variation in adult nutrition affects testes mass in hemimetabolous insects. With millions of hemimetabolous species on this planet, the lack of studies on testes investment has been a notable omission (*for holometabola, see* Ward and Simmons 1991; Droney 1998).

Interestingly, we did not find evidence that inbreeding affected sperm traits or testes mass for males in this population. These results are inconsistent with theoretical and empirical studies showing negative effects of inbreeding on male reproductive traits (Gage et al. 2006; Asa et al. 2007; Fitzpatrick and Evans 2009; Weeks et al. 2009). Our study suggests that typical levels of inbreeding might not always have negative effects on sperm traits (Gomendio et al. 2000; van Eldik et al. 2006; Zajitschek et al. 2009; Mehlis et al. 2012; Johnson et al. 2015), even when paired with nutritional stress.

We found that adult males had reduced testes mass when they experienced inferior adulthood nutrition. Adult age also had an effect on testes mass and sperm traits, as shown in other species (*reviewed in* Johnson and Gemmell 2012). Previous work in Hawaiian *Drosophila,* rats, and mallard ducks has shown that testis size is dependent on the acquisition and allocation of resources derived from both juvenile (Edmonds et al. 2003) and adult diets (Droney 1998; Butler et al. 2013). Testis size is a critical male reproductive trait linked to sperm competition and ejaculate expenditure (Hosken and Ward 2001; Parker and Ball 2005; *but see* Kelly 2008). Larger testis size can be positively correlated with sperm production (Møller 1988, 1989; Svärd and Wiklund 1989; Gage 1994; Stockley et al. 1997) and subsequently increased reproductive success (Preston et al. 2003).

Cactus fruit is readily available for *N. femorata* through the spring and summer months, but then becomes less abundant when approaching the winter months (Gillespie et al. 2014). The decreased availability of fruit may limit male physical condition and lead to the patterns we see here. Nonexclusively, the absence of fruit may serve as a cue that there are limited fecund females in the area, and so males should invest less into sexual traits and invest more into other life‐history traits until more high‐quality resources return. Indeed, relatively few offspring are produced in winter months in natural populations (L.A. Cirino, *in prep*), suggesting males should decrease investment in sexual traits under fruit‐limited conditions. Males in multiple animal species experience testis regression in response to cues indicating limited reproductive opportunities (*reviewed in* Young and Nelson 2001). It will be exciting to test further the effects of seasonal change on sexual traits in *N. femorata*.

We did not find that adult diet quality had a significant effect on either sperm concentration or viability. These results were inconsistent with other studies which have found that nutritional stress can impair sperm traits and performance (Gage and Cook 1994; McGraw et al. 2007; Rahman et al. 2014). The onset, duration, and extent of dietary restriction are all known to influence the magnitude of effects on sperm traits (Amitin and Pitnick 2007; Engqvist 2008; Fricke et al. 2008). Males in this experiment were sacrificed between 21 and 73 days in age, and yet, we did not find any evidence that older individuals suffered impaired sperm traits and performance as linked to nutrition. Additionally, aspects of spermatogenesis and male sexual inactivity during the study may have affected the results related to sperm traits. All juveniles in our study had access to the high‐quality diet until adult eclosion. This excellent nutrition may have come at the crucial time for the development of sperm traits, as spermatogenesis initiates during the penultimate and ultimate juvenile instars in other hemipterans (Economopoulos and Gordon 1971; Brent 2010). Even if adult nutrition negatively affected sperm traits, the males in our study could have generated a supply of mature sperm in their seminal vesicles that was not depleted, even as testes apparently shrank (our males were housed alone, without access to females). Future studies should test this hypothesis by providing nutritionally deprived males access to receptive females, then measuring sperm traits.

Why were the primary sexual traits of male *N. femorata* not affected by inbreeding? It is possible that there was a small inbreeding effect, and we needed more power to reveal it. Also, limited inbreeding combined with the history of our population could have together, or separately, been responsible. Our study individuals were subjected to only one generation of inbreeding. A single generation of inbreeding can be sufficient to cause negative effects on sperm competitiveness (Simmons 2011) and other ejaculate traits (Malo et al. 2010; Fox et al. 2012) in some cases. Yet, many previous studies have used multiple generations of inbreeding, which may have amplified detrimental effects (Konior et al. 2005; Michalczyk et al. 2010). Importantly, our study population may be robust to natural low levels of inbreeding, given its history. This species was introduced to Florida in the 1950s, most likely on cacti nursery stock (Baranowski and Slater 1986). Thus, the population may have experienced inbreeding in the past that could have purged reproductively deleterious alleles. It would be interesting to examine other populations of *N. femorata* in their native range to test for similar effects. It is worth noting that *N. femorata* in this study may have suffered detrimental effects of inbreeding in traits we did not measure here, such as survivorship.

In conclusion, our work suggests that while adult nutrition had overt negative effects on testes growth, realistically low levels of inbreeding did not. Additionally, neither adult nutrition quality nor inbreeding had any detectable effects on sperm concentration and viability. Future research should elucidate the interactions between seasonality, natural dietary fluctuations, and plastic changes to male reproductive traits. Previous research suggests that the timing of reproductive events is mediated by seasonal cues (Rubenstein and Wikelski 2003; Brown and Shine 2006). It is possible that males are able to strategically reallocate resources away from other primary sexual traits, including accessory glands, in response to seasonal cues indicating suboptimal resource availability and, subsequently, reduced reproductive opportunities. This plasticity in sexual trait investments may also relate to changes in social environment. Males are able to manipulate the quality of ejaculates in response to changes in sperm competition risk and intensity (Wedell et al. 2002). This phenomenon may also occur with other male primary sexual traits for lengthier sperm competitive cues. Further research on this topic is greatly needed.

## Conflict of Interest

None declared.
